# The Association Between Periodontal Disease and Polycystic Ovary Syndrome: A Systematic Review and Meta-Analysis

**DOI:** 10.3390/dj13050188

**Published:** 2025-04-25

**Authors:** Vincenzo Bitonti, Tiziana Perri, Lorenzo Cigni, Domenico Familiari, Giuseppe Vazzana, Rocco Franco

**Affiliations:** 1 UOC Odontostomatologia e Chirurgia Orale, AUSL Modena, 41122 Modena, Italy; v.bitonti@ausl.mo.it (V.B.); l.cigni@ausl.mo.it (L.C.); d.familiari@ausl.mo.it (D.F.); g.vazzana@ausl.mo.it (G.V.); 2 UO Ginecologia e Ostetricia Ospedale SS Annunziata, AUSL Ferrara, 44121 Ferrara, Italy; t.perri@ausl.fe.it

**Keywords:** periodontal disease, polycystic ovary syndrome, systemic inflammation, insulin resistance, PRISMA, systematic review

## Abstract

**Background:** Periodontal disease (PD) and polycystic ovary syndrome (PCOS) are two prevalent conditions that have been independently associated with systemic inflammation and hormonal dysregulation. Emerging evidence suggests a potential bidirectional relationship between these conditions, but the nature and strength of this association remain unclear. **Objective:** This systematic review aimed to evaluate and synthesize the existing evidence on the association between periodontal disease and polycystic ovary syndrome, following the Preferred Reporting Items for Systematic Reviews and Meta-Analyses (PRISMA) guidelines. **Methods:** A comprehensive literature search was conducted in multiple databases, including PubMed, Scopus, and Web of Science, from 1990 to 2025. Studies investigating the association between PD and PCOS in terms of prevalence, severity, and shared pathophysiological mechanisms were included. Two independent reviewers screened studies for eligibility, extracted data, and assessed methodological quality using validated tools. Discrepancies were resolved through consensus. Meta-analysis was conducted where appropriate. **Results:** A total of nine studies were included. Most studies reported a higher prevalence and severity of periodontal disease among women with PCOS compared to controls. Shared mechanisms, including systemic inflammation, insulin resistance, and hormonal dysregulation, were frequently proposed as underlying factors. However, heterogeneity in study designs, diagnostic criteria, and confounding adjustments limited the comparability of findings. **Conclusions:** This systematic review supports a potential association between periodontal disease and polycystic ovary syndrome, likely mediated by common inflammatory and metabolic pathways. However, the evidence is limited by heterogeneity and methodological biases. Further well-designed longitudinal studies are needed to clarify causal relationships and explore the clinical implications of integrating periodontal health management into PCOS care.

## 1. Introduction

Polycystic ovary syndrome (PCOS) is one of the most prevalent endocrine disorders affecting women of reproductive age, characterized by a constellation of symptoms, including menstrual irregularities, hyperandrogenism, and polycystic ovarian morphology. With an estimated prevalence of 5% to 20% worldwide, depending on diagnostic criteria, PCOS significantly impacts women’s quality of life and is associated with a broad spectrum of metabolic and reproductive complications [1,2]. PCOS is a common endocrine disorder that affects women of reproductive age, with a global prevalence ranging from 5% to 20%, depending on diagnostic criteria. It is a complex condition characterized by hormonal imbalances, metabolic dysfunction, and reproductive abnormalities. PCOS is associated with a wide range of symptoms, including irregular menstrual cycles, hyperandrogenism (excess male hormones leading to symptoms like hirsutism, acne, and hair loss), and polycystic ovarian morphology observed on ultrasound. However, its manifestations can vary significantly among individuals.

According to the World Health Organization (WHO) and the National Institute of Child Health and Human Development (NICHD), PCOS is one of the leading causes of infertility in women of reproductive age, affecting 8–13% of women worldwide, with 70% of affected women remaining undiagnosed. One diagnostic method for this syndrome is the Rotterdam criteria, which utilizes clinical or biochemical evidence of hyperandrogenism, evidence of oligo-anovulation, and ultrasonographic identification of polycystic ovary morphology [3]. These complications include insulin resistance, type 2 diabetes mellitus, cardiovascular disease, infertility, and psychological distress. Increasingly, evidence has highlighted that PCOS is not solely a reproductive disorder but a systemic condition with far-reaching implications beyond the reproductive system. Similarly, periodontal disease (PD), a chronic inflammatory condition affecting the supporting structures of the teeth, is a major global public health concern. Periodontitis is a chronic multifactorial inflammatory disease, which, similarly to PCOS, is associated with chronic low-grade inflammation. Furthermore, inflammatory markers such as C-reactive protein (CRP), interleukin-6 (IL-6), and tumor necrosis factor-α (TNF-α) are elevated in both conditions, which could influence wound healing and tissue regeneration in periodontal therapy [4]. It encompasses two primary forms: gingivitis, a reversible inflammation of the gingiva, and periodontitis, a more severe and irreversible destruction of the periodontal ligament and alveolar bone. Periodontitis, if left untreated, may lead to tooth loss and has been increasingly recognized for its systemic effects, including associations with cardiovascular disease, diabetes, adverse pregnancy outcomes, and other inflammatory conditions [5,6].

Recent research has suggested that periodontal disease and PCOS share common pathophysiological mechanisms, particularly chronic low-grade inflammation and hormonal imbalances. Inflammatory markers such as CRP, IL-6, and TNF-α are elevated in both conditions, reflecting a state of systemic inflammation that may serve as a biological link between them. Moreover, insulin resistance—a hallmark feature of PCOS—has been implicated in the pathogenesis of periodontal disease, as hyperglycemia and advanced glycation end products can exacerbate periodontal tissue destruction [7]. Conversely, the chronic inflammation associated with periodontal disease may worsen insulin sensitivity, creating a bidirectional relationship between the two conditions [8]. Hormonal factors, particularly elevated androgen levels in women with PCOS, may also contribute to an increased susceptibility to periodontal disease by influencing immune responses and tissue integrity [9,10,11,12,13,14].

The clinical overlap between periodontal disease and PCOS has spurred interest in understanding whether a direct association exists and, if so, the implications for patient care. Observational studies have reported a higher prevalence and severity of periodontal disease in women with PCOS compared to healthy controls. These findings have raised questions about whether periodontal disease might be considered a comorbidity of PCOS or even a potential risk factor influencing its clinical course. Given the established impact of periodontal health on systemic conditions, exploring the relationship between PD and PCOS could open new avenues for interdisciplinary approaches to patient management. For instance, integrating periodontal health assessments and interventions into the care of women with PCOS could offer a cost-effective strategy to mitigate systemic inflammation and improve overall health outcomes [15,16,17].

Despite the growing body of literature on this topic, significant gaps remain in understanding the association between periodontal disease and PCOS [18]. The heterogeneity in diagnostic criteria for both conditions, as well as variations in study designs, population characteristics, and methods of assessing periodontal status, further complicates the synthesis of findings. Additionally, the influence of potential confounders such as age, body mass index (BMI), smoking status, and socioeconomic factors on the observed associations has not been consistently addressed [19].

This systematic review aims to provide a comprehensive synthesis of the current evidence on the association between periodontal disease and polycystic ovary syndrome, following the Preferred Reporting Items for Systematic Reviews and Meta-Analyses (PRISMA) guidelines. By critically evaluating the existing literature, this review seeks to address key questions: (1) Is there a significant association between PD and PCOS in terms of prevalence and severity? (2) To what extent do methodological limitations in the existing studies impact the validity of the findings? (3) What are the clinical implications of this association for the management of women with PCOS? In doing so, this review aims to contribute to the growing field of research on the interplay between systemic and oral health and to highlight areas for future investigation. Understanding the association between PD and PCOS is not only of academic interest. By identifying potential shared mechanisms and bidirectional influences, this review seeks to inform clinical practice and guide strategies for integrated care that address both systemic and oral health in women with PCOS.

## 2. Materials and Methods

### 2.1. Study Design

This systematic review aimed to evaluate the connection between periodontal disease and polycystic ovary syndrome. The review followed the Preferred Reporting Items for Systematic Reviews and Meta-Analyses (PRISMA) guidelines. A comprehensive search strategy was employed to gather relevant studies, followed by strict eligibility criteria for the inclusion and exclusion of studies.

### 2.2. Eligibility Criteria

We created a PECO to meet the purpose of the systematic review.

Participants: The participants are people.

Exposure: The patients with PCOS.

Comparison: The patients are healthy.

Outcome: The aim is to assess the prevalence and correlation between PCOS and periodontal disease.

Inclusion Criteria:

Randomized controlled trials (RCTs), clinical trials, cohort studies, and observational studies;

Studies evaluating periodontal health among PCOS patients and healthy patients;

Articles published in peer-reviewed journals;

Studies reporting on at least one of the following outcomes: probing depth, clinical attachment level, bleeding on probing, gingival bleeding index, and plaque index;

Full-text articles published in English.

Exclusion Criteria:

Non-clinical studies (e.g., laboratory, animal studies);

Studies focusing on diseases or conditions unrelated to periodontal disease;

Studies with incomplete data on bromelain dosage, administration, or outcome measures;

Abstracts, reviews, or editorials.

### 2.3. Information Sources

A systematic search was conducted across multiple electronic databases, including:

PubMed;

Scopus;

Web of Science.

The search period covered from the inception of the databases from 1990 until January 2025.

### 2.4. Search Strategy

The following key terms were used in different combinations: “Polycystic ovary Syndrome” and “Periodontal Disease”. Boolean operators such as AND, OR, and NOT were applied to refine the search results.

### 2.5. Study Selection

Two independent reviewers screened the search results using the eligibility criteria. Titles and abstracts were reviewed first, followed by a full-text evaluation of potentially eligible studies. Discrepancies between the two reviewers were resolved by consensus or consultation with a third reviewer.

### 2.6. Data Extraction

Data extraction was carried out by two independent reviewers using a standardized data extraction form. The following data were extracted: name, study design, population, key results, inclusion/exclusion criteria, and primary outcome.

### 2.7. Quality Assessment

In this review, the Risk of Bias in Non-randomized Studies of Interventions (ROBINS-E) tool was employed to evaluate potential biases in the included studies. This tool offers a structured approach for assessing bias in non-randomized research. Each study underwent an independent evaluation by at least two reviewers, trained in the ROBINS-E methodology and followed established guidelines. The assessment covered seven key domains: confounding, participant selection, intervention classification, deviations from intended interventions, missing data, outcome measurement, and selective reporting of results. To ensure consistency and objectivity, any disagreements among reviewers were resolved through discussion and consensus. If consensus could not be achieved, a third reviewer was consulted for the final decision. The application of the ROBINS-E tool provided a thorough assessment of bias, highlighting both the strengths and limitations of the included studies. This process contributes to a more accurate evaluation of the quality and reliability of the evidence, allowing for better-informed conclusions. Additionally, to assess potential publication bias, gb version 4.3 was utilized to generate a funnel plot, incorporating the Egger test.

### 2.8. Statistical Analysis

The pooled analyses were performed using the software Review Manager version 5.2.8 (Cochrane Collaboration, Copenhagen, Denmark; 2014). Periodontal parameters were analyzed between two groups: PCOS and healthy. The parameters of all items were examined, and those that had something in common were correlated. Specifically, plaque index, probing depth, gingival index, plaque index, and clinical attachment loss were analyzed. The function of continuous variables and standard deviation was used. A random effects model was used given the high heterogeneity among the studies. Heterogeneity among studies was evaluated using the Higgins Index (I2) and the Chi-squared test and classified as follows: low heterogeneity (<30%), medium heterogeneity (30–60%), and high heterogeneity (>60%).

### 2.9. Grade of Strength

We applied the Grading of Recommendations Assessment, Development and Evaluation (GRADE) ranking system to measure the quality of evidence and determine the level of certainty for the results of this review [20].

## 3. Results

### 3.1. Study Characteristics

**Figure 1:**
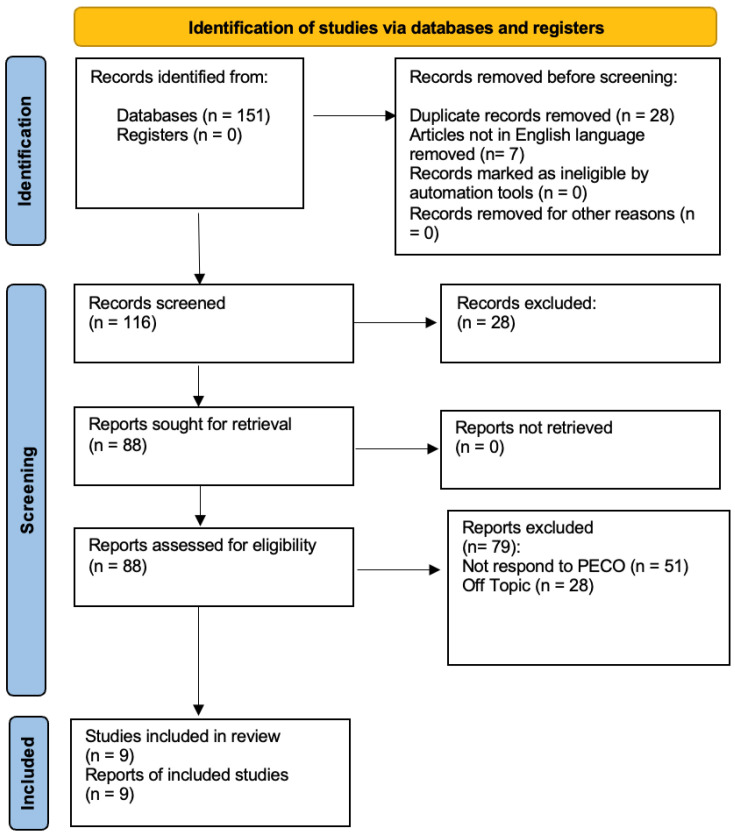
Prisma Flowchart.

At the conclusion of the research, a total of 151 studies were identified through searches across three databases. In the initial phase, 28 duplicates were removed, along with seven studies that were not in English. Specifically, one study was excluded from PubMed, three from Web of Science, and three from Scopus. During the preliminary screening, 28 articles were excluded for being systematic reviews, as they did not meet the inclusion criteria. In the final screening stage, the abstracts and full texts of 88 articles were reviewed, but only 9 were selected for inclusion in this systematic study, as illustrated in the PRISMA 2020 flowchart (Figure 1). A total of 79 studies were excluded—51 for not meeting the PECO criteria and 28 for being unrelated to the research topic. The remaining studies were selected based on title and abstract screening following the PECO model. Ultimately, nine studies were included in the final publication. The selected studies were conducted between 2011 and 2014 in different regions, including Taiwan and Italy, analyzing a total of 208 participants.

The first study, titled Impact of Body Mass Index and PCOS Subtypes on Periodontal Health in Chinese Women with PCOS and Periodontitis, conducted a retrospective case–control analysis involving 88 women with PCOS and periodontitis, compared to 82 healthy controls matched for age and BMI. The results revealed that women with PCOS exhibited a significantly higher gingival bleeding index (GBI), indicative of more severe gingival inflammation (*p* < 0.05). Probing depth (PD) and the proportion of sites with PD ≥ 5, PD ≥ 6, and PD ≥ 7 mm did not differ significantly between the two groups (*p* > 0.05). Among the PCOS cohort, 77.27% were classified as Type I, 9.09% as Type II, 9.09% as Type III, and 4.55% as Type IV. Androstenedione levels showed significant variation across these subtypes (*p* < 0.05). Notably, individuals with a BMI ≥ 24.0 kg/m^2^ exhibited higher GBI values alongside a distinct periodontal profile compared to those with a BMI < 24.0 kg/m^2^ (*p* < 0.05), underscoring the influence of obesity on periodontal outcomes [21].

The second study, Subgingival Microflora in Adolescent Females with PCOS and Its Association with Oral Hygiene, Gingivitis, and Selected Metabolic and Hormonal Parameters, investigated the subgingival microflora in 32 adolescent females with PCOS and 23 healthy controls. Despite comparable plaque index (PLI) and gingival index (GI) scores between the two groups (*p* > 0.05), with GI values indicating mild gingivitis, distinct microbial differences emerged. Elevated levels of pathogenic bacteria such as Porphyromonas gingivalis, Fusobacterium nucleatum, and Tannerella forsythia were observed in the PCOS group. These microbial changes are correlated with hormonal abnormalities, including elevated testosterone levels and fasting insulin levels. The study highlighted a weak but significant correlation between red complex bacterial counts and PLI (*p* < 0.05), linking hormonal dysregulation with microbial imbalances that may contribute to periodontal inflammation [22].

The third study, Periodontal Disease in Polycystic Ovary Syndrome, focused on 25 non-obese PCOS patients compared to 27 age- and weight-matched controls. The findings indicated significantly worse periodontal outcomes in the PCOS group, with higher gingival index (GI) scores of 1.6 ± 0.5 compared to 1.2 ± 0.4 in the controls (*p* = 0.002) and an increased probing depth (PD) of 2.8 ± 0.4 mm versus 2.4 ± 0.3 mm (*p* = 0.013). Bleeding on probing (BOP%) was also notably higher in PCOS patients at 45.3%, compared to 29.1% in controls (*p* = 0.001). Moreover, gingival crevicular fluid (GCF) volume, a subclinical marker of inflammation, was significantly elevated in the PCOS group. Oxidative stress markers, including myeloperoxidase (MPO) and nitric oxide (NO) in GCF, were also markedly higher (*p* = 0.019 and *p* = 0.02, respectively), suggesting a pro-inflammatory state localized to the gingival tissues [23].

The fourth study, Association Between PCOS, Oral Microbiota, and Systemic Antibody Responses, divided participants into four groups: PCOS patients with and without gingivitis and systemically healthy controls with and without gingivitis. The study demonstrated that PCOS patients with gingivitis exhibited significantly higher levels of periodontal pathogens such as *P. gingivalis*, *F. nucleatum*, and *T. forsythia*. Probing depth (PD) and bleeding on probing (BOP) scores were also significantly elevated in this group (*p* < 0.05). Furthermore, systemic antibody responses to these bacteria were heightened in PCOS patients, particularly those with gingivitis, suggesting an interplay between systemic inflammation and periodontal disease [24].

The fifth study, Comparative Assessment of Periodontal Status in Subjects With and Without PCOS and Its Correlation With BMI included 30 women with PCOS and 30 healthy controls. The PCOS patients exhibited worse periodontal health, with a mean plaque index (PI) score of 0.62 ± 0.54 compared to 0.04 ± 0.05 in controls (*p* < 0.001) and a periodontal disease index (PDI) score of 0.60 ± 0.40 versus 0.07 ± 0.10 (*p* < 0.001). Probing depth (PD) was slightly higher in the PCOS group at 1.28 ± 0.23 mm compared to 1.18 ± 0.14 mm in controls (*p* < 0.05). BMI was significantly higher in the PCOS group at 25.63 ± 3.64 compared to 23.33 ± 3.91 (*p* < 0.05), and increased BMI correlated with worsening periodontal parameters [25].

The sixth study, Association Between Metabolic and Hormonal Profiles, Proinflammatory Cytokines in Saliva, and Gingival Health in Adolescent Females with PCOS, examined 31 adolescent females with PCOS and 28 healthy controls. Salivary cytokines, including IL-6, IL-1β, and TNF-α, were significantly elevated in the PCOS group (*p* < 0.001), correlating with increased gingival index (GI) scores and bleeding on probing (BOP%). Salivary testosterone levels were also higher in the PCOS group (*p* = 0.0007) and inversely correlated with gingival inflammation markers, suggesting an interaction between hormonal dysregulation and inflammatory responses in periodontal tissues [26].

The seventh study, Comparison of Prevalence of Periodontal Disease in Women with PCOS and Healthy Controls, assessed 98 PCOS patients and 98 controls. The prevalence of periodontal disease was significantly higher in the PCOS group, with clinical attachment loss (CAL) averaging 1.5 ± 0.6 mm compared to 1.1 ± 0.4 mm in controls (*p* < 0.05). Probing depth (PD) was also greater in the PCOS group at 2.9 ± 0.7 mm versus 2.4 ± 0.5 mm (*p* < 0.05), emphasizing PCOS as an independent risk factor for periodontal disease [27].

The eighth study, Evaluation of Periodontal Status in Women with PCOS Versus Healthy Women: A Cross-Sectional Study, demonstrated higher gingival index (GI) scores of 1.8 ± 0.4 in PCOS patients compared to 1.2 ± 0.3 in controls (*p* < 0.05), and increased probing depths of 2.7 ± 0.5 mm versus 2.3 ± 0.4 mm (*p* < 0.05). These differences were strongly associated with elevated androgen levels in the PCOS group, linking systemic hormonal imbalances with periodontal inflammation [28].

The ninth study, Gingival Inflammation and Leukocyte–Endothelial Cell Interactions in Women with PCOS, highlighted heightened neutrophil activation and leukocyte-endothelial interactions in PCOS patients, contributing to gingival inflammation. Elevated gingival crevicular fluid (GCF) volumes, coupled with increased probing depths (PD) and bleeding on probing (BOP%), were significantly correlated with androgen levels and markers of insulin resistance, revealing the systemic inflammatory underpinnings of periodontal disease in PCOS [29] (Table 1 and Table 2).

### 3.2. Meta-Analysis

The meta-analysis was conducted using a random model effect because of the high heterogeneity between all parameters.

**Figure 2:**
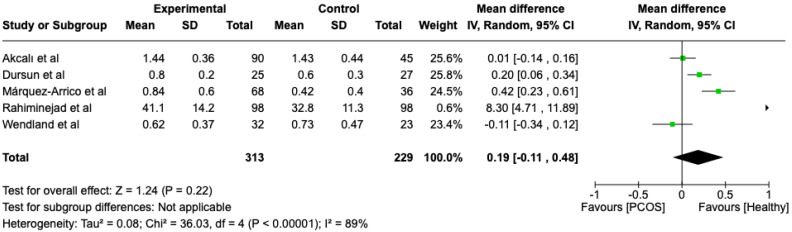
Forest plot regarding differences in plaque index scores between the PCOS and healthy groups. Green: studies included; black: the mean difference of all studies [22,23,24,27,29].

The first parameter to be studied and analyzed was the plaque index. We compared the PCOS and Healthy groups regarding this periodontal parameter. The analysis showed high heterogeneity (I2 = 89%). The study analyzed the differences in plaque index scores between patients with PCOS and healthy. The overall effect, reported in the forest plot (Figure 2), revealed that the two groups did not have a difference in terms of plaque index scores; the healthy group had a good plaque index score with respect to the PCOS group (Mean 0.19; Mean Difference: −0.11;0.48; Z = 1.24; *p* = 0.22).

**Figure 3:**
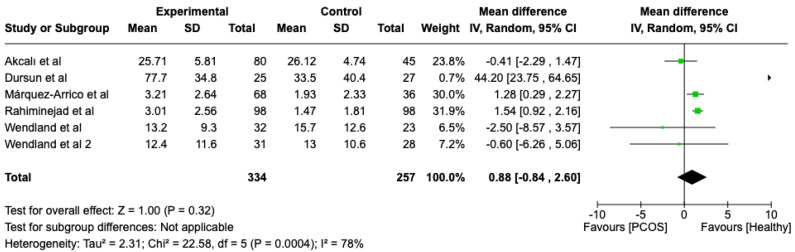
Forest plot regarding differences in bleeding on probing between the PCOS and healthy groups. Green: studies included; black: the mean difference of all studies [22,23,24,26,27,29].

We compared the PCOS and Healthy groups regarding bleeding on probing. The analysis showed high heterogeneity (I2 = 78%). The study analyzed the differences regarding bleeding on probing between patients with PCOS and healthy. The overall effect, reported in the forest plot (Figure 3), revealed that the two groups did have a different statistical significance in terms of bleeding on probing; the healthy group had good bleeding on probing with respect to the PCOS group (Mean 0.88; Mean Difference: −0.84;2.60; Z = 1.00; *p* = 0.00004).

**Figure 4:**
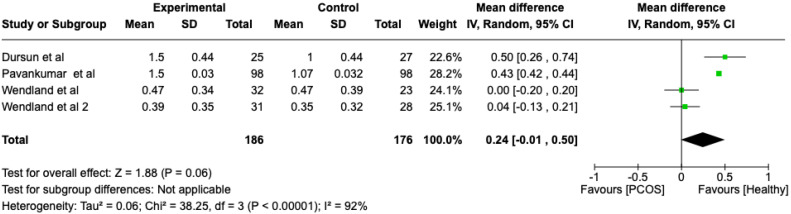
Forest plot regarding differences in gingival index scores between the PCOS and healthy groups. Green: studies included; black: the mean difference of all studies [22,23,26,28].

We compared the PCOS and Healthy groups regarding the gingival index scores. The analysis showed high heterogeneity (I2 = 92%). The study analyzed the differences regarding the gingival index scores between patients with PCOS and healthy. The overall effect, reported in the forest plot (Figure 4), revealed that the two groups did have a different statistical significance in the gingival index scores; the healthy group had a good gingival index score with respect to the PCOS group (Mean 0.24; Mean Difference: −0.01;2.60; Z = 1.88; *p* = 0.00001).

**Figure 5:**
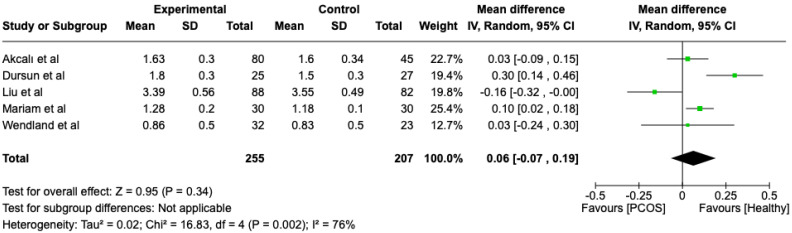
Forest plot regarding differences in probing depth between the PCOS and healthy groups. Green: studies included; black: the mean difference of all studies [21,22,23,24,25].

We compared the PCOS and Healthy groups regarding probing depth. The analysis showed high heterogeneity (I2 = 76%). The study analyzed the differences regarding probing depth between patients in the PCOS and healthy groups. The overall effect, reported in the forest plot (Figure 5), revealed that the two groups did not have a different statistical significance in the probing depth; the healthy group had a good probing depth with respect to the PCOS group but without statistical significance (Mean 0.06; Mean Difference: −0.07; 0.19; Z = 0.95; *p* = 0.34).

**Figure 6:**
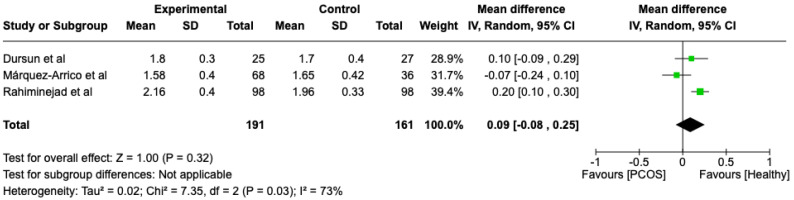
Forest plot regarding differences in clinical attachment loss between the PCOS and healthy groups. Green: studies included; black: the mean difference of all studies [23,27,29].

We compared the PCOS and Healthy groups regarding clinical attachment loss. The analysis showed high heterogeneity (I2 = 73%). The study analyzed the differences regarding clinical attachment loss between patients in the PCOS and healthy groups. The overall effect, reported in the forest plot (Figure 6), revealed that the two groups did not have a different statistical significance in the clinical attachment loss; the healthy group had a good clinical attachment loss with respect to the PCOS group but without statistical significance (Mean 0.09; Mean Difference: −0.08;0.25; Z = 1; *p* = 0.32).

Across all studies, women with PCOS demonstrated significantly worse periodontal health compared to healthy controls. Higher values of GI, PD, and BOP were observed in PCOS patients, indicating greater levels of gingival inflammation and periodontal tissue destruction. One study reported a significantly increased GCF (gingival crevicular fluid) volume in PCOS patients, suggesting heightened localized inflammation.

The meta-analysis further confirmed that PCOS patients had a statistically significant increase in gingival inflammation (GI: I^2^ = 92%, *p* = 0.00001) and BOP (I^2^ = 78%, *p* = 0.00004). However, differences in plaque index and clinical attachment loss were not statistically significant (*p* = 0.22 and *p* = 0.32, respectively). These findings suggest that while PCOS is associated with increased gingival inflammation, additional factors such as oral hygiene habits, diet, and BMI may influence overall periodontal disease progression.

Black: the mean difference of all studies

### 3.3. Quality Assessment and Risk of Bias of Included Articles

**Figure 7:**
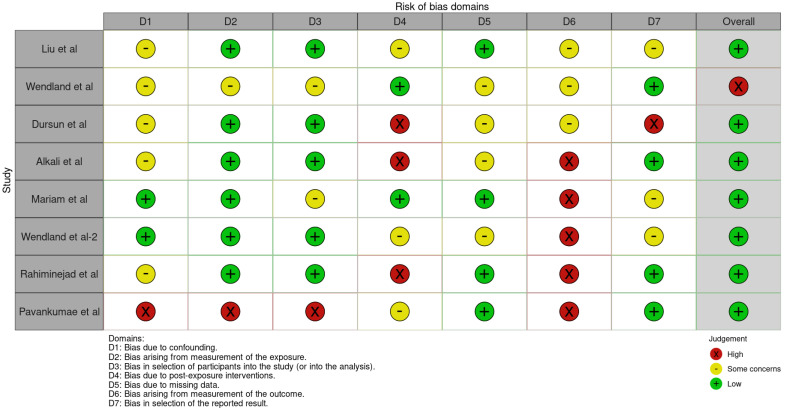
ROBINS-E of the study selected for this meta-analysis [21,22,23,24,25,25,27,28,29].

**Figure 8:**
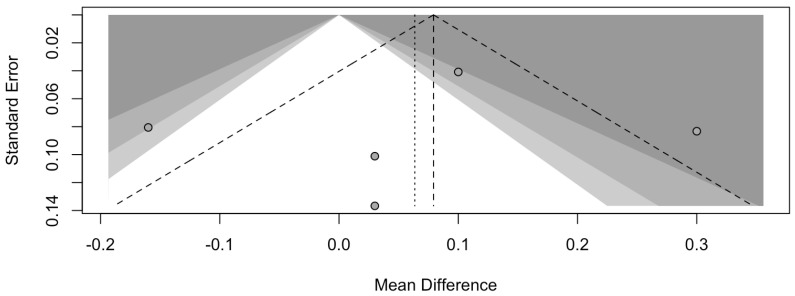
Funnel plot of studies comparing plaque index scores. The plot shows an asymmetry with regard to the studies.

The risk of bias in the included studies is reported in Figure 7. Regarding the bias due to confounding, most studies have some concerns. The bias arising from measurement is a parameter with a low risk of bias. Many studies have a low risk of bias due to bias in the selection of participants. Bias due to post-exposure cannot be calculated due to high heterogeneity. The bias due to missing data is low in many studies. Bias arising from the measurement of the outcome is low. Bias in the selection of the reported results is high in most studies. The final results show that four studies have a low risk of bias. Table 3 evaluates the degree of evidence, and therefore, we can say that the evidence of the results is high, however the heterogeneity of the studies. To mediate the R study, studies involving the plaque index were considered with regard to intercept bias. The analysis showed the presence of publication bias, especially regarding studies with a smaller number of patients (Figure 8).

## 4. Discussion

The intricate relationship between polycystic ovary syndrome (PCOS) and periodontal disease represents a convergence of systemic and localized inflammatory processes, hormonal imbalances, and metabolic dysregulation. The findings of this review consistently reveal that women with PCOS exhibit worse periodontal outcomes compared to healthy controls. These outcomes include increased gingival inflammation, deeper probing depths, higher plaque indices, and a greater prevalence of clinical attachment loss (CAL). The systemic hormonal and metabolic abnormalities in PCOS provide a plausible mechanism linking the condition to periodontal disease. Hyperandrogenism, a hallmark of PCOS, appears to play a central role in modulating the inflammatory milieu. Elevated levels of androgens such as testosterone and androstenedione have been associated with increased expression of pro-inflammatory cytokines, including interleukin-6 (IL-6) and tumor necrosis factor-alpha (TNF-α), in gingival tissues. These cytokines are key mediators of the inflammatory response and contribute to the breakdown of periodontal tissues. Moreover, the insulin resistance commonly observed in PCOS exacerbates systemic inflammation, creating a pro-inflammatory state that amplifies periodontal disease progression [30,31,32,33].

The microbiological data from several studies further strengthen the association between PCOS and periodontal disease. Women with PCOS harbor higher levels of periodontal pathogens, such as *Porphyromonas gingivalis*, *Tannerella forsythia*, and *Fusobacterium nucleatum*, which are well-established contributors to periodontal tissue destruction. These pathogenic bacteria thrive in the dysbiotic oral environment influenced by hormonal fluctuations in PCOS. Additionally, systemic antibody responses to these pathogens are heightened in PCOS, suggesting a broader immune activation that could further exacerbate periodontal inflammation [34,35,36,37,38,39]. Obesity, a common comorbidity in PCOS, acts as an additional layer of complexity. A higher body mass index (BMI) is independently associated with worse periodontal outcomes due to the release of adipokines and pro-inflammatory cytokines from adipose tissue. These molecules further contribute to systemic inflammation and impair immune function, compounding the risk of periodontal disease in women with PCOS.

However, it is noteworthy that periodontal inflammation is also present in non-obese PCOS patients, indicating that PCOS itself, independent of obesity, is a risk factor for periodontal disease [40,41,42,43]. Despite the robust evidence linking PCOS and periodontal disease, several limitations must be acknowledged. Many studies included in this review were cross-sectional, limiting the ability to establish causal relationships. Our results are in line with the results obtained by Kellesarian, who carried out a systematic review similar to ours regarding the association between PD and PCOS. However, our review performed a meta-analysis regarding periodontal parameters (GI, CAL, PD, etc.), comparing them with a control group. In contrast, Kellesarian’s review did not evaluate these periodontal parameters; it only evaluated the parameters of pro-inflammatory cytokines in crevicular fluid and the count of periodontopathogenic bacteria, which our meta-analysis did not evaluate [44]. Differences in study populations, diagnostic criteria for PCOS and periodontal disease, and methodologies for assessing periodontal health introduce heterogeneity, which may influence the interpretation of results. Additionally, the role of confounding factors, such as diet, stress, and oral hygiene practices, warrants further investigation to isolate the effects of PCOS on periodontal health.

### 4.1. Clinical Implications

One of the key clinical implications is the need for interdisciplinary collaboration between gynecologists, endocrinologists, and dental professionals. PCOS management typically focuses on metabolic and reproductive health, but our findings suggest that routine periodontal assessment and early intervention could help mitigate systemic inflammation and its potential impact on PCOS severity. Gynecologists and endocrinologists should consider including oral health evaluations as part of PCOS patient assessments, while periodontists and dentists should be made aware of the increased risk of periodontal disease in women with PCOS. Healthcare providers should educate PCOS patients on the importance of maintaining good periodontal health and encourage routine dental visits as part of their overall health management. Simple interventions, such as regular dental check-ups, patient education on proper oral hygiene, and early periodontal treatment, could help reduce the inflammatory burden in these patients. Furthermore, lifestyle modifications, including dietary changes and weight management—already recommended for PCOS—should be reinforced with an emphasis on their role in supporting periodontal health.

### 4.2. Limitations of This Review

One of the primary limitations of this systematic review is the high heterogeneity observed across the included studies, with I^2^ values ranging from 73% to 92%. This variability stems from differences in study designs, diagnostic criteria for both PCOS and periodontal disease, sample sizes, and population characteristics. Such heterogeneity limits the ability to draw definitive conclusions and reduces the generalizability of our findings. Additionally, the small number of included studies (n = 9) further compounds this issue, as a limited dataset can amplify variability and restrict the reliability of pooled estimates. Another notable limitation is the geographical concentration of studies, with most originating from Taiwan and Italy, which may not adequately represent global populations. Future research should focus on including more diverse cohorts to enhance the external validity of findings. Furthermore, while this review touches on clinical implications, a more integrated approach to incorporating periodontal health into PCOS management should be explored in future studies. Given these limitations, caution is necessary when interpreting the results, and additional well-designed, multicenter studies are required to strengthen the evidence base.

## 5. Conclusions

The evidence presented in this review underscores the significant association between polycystic ovary syndrome (PCOS) and periodontal disease. PCOS is characterized by systemic hormonal and metabolic disturbances, contributing to an increased risk of periodontal inflammation and tissue destruction. Elevated androgen levels, systemic inflammation, and microbiological dysbiosis collectively form the pathophysiological basis for this association.

The bidirectional nature of the relationship between systemic and periodontal health highlights the need for integrated care approaches in the management of women with PCOS. Periodontal assessments should be incorporated into routine care for PCOS patients, with an emphasis on early detection and intervention to mitigate oral and systemic complications. Interdisciplinary collaboration among gynecologists, endocrinologists, and periodontists is essential to provide holistic care that addresses systemic and oral health.

Future research should focus on longitudinal studies to elucidate the causal pathways linking PCOS and periodontal disease, as well as randomized controlled trials to evaluate the effectiveness of targeted interventions. Investigating the impact of hormonal therapy, anti-inflammatory agents, and lifestyle modifications on periodontal outcomes in women with PCOS could provide valuable insights for improving clinical management.

By acknowledging the systemic nature of PCOS and its implications for oral health, healthcare providers can develop comprehensive strategies to enhance the quality of life for affected women, addressing both their reproductive and periodontal health needs.

## Figures and Tables

**Table 1:** Summary and explanation by table of the main studies in this meta-analysis.

Title	Study Design	Population	Key Results
Impact of Body Mass Index and PCOS Subtypes on Periodontal Health in Chinese Women with PCOS and Periodontitis [21]	Retrospective case–control	88 PCOS patients, 82 controls	Higher GBI in PCOS (*p* < 0.05); BMI ≥24 kg/m^2^ linked to distinct profiles.
Subgingival Microflora in Adolescent Females with PCOS and Its Association with Oral Hygiene, Gingivitis, and Selected Metabolic and Hormonal Parameters [22]	Observational	32 PCOS adolescents, 23 controls	Elevated *P. gingivalis*, *F. nucleatum*; correlation with hormonal levels.
Periodontal Disease in Polycystic Ovary Syndrome [23]	Comparative case–control	25 non-obese PCOS, 27 controls	Higher GI, PD, BOP%; elevated GCF MPO and NO in PCOS (*p* < 0.05).
Association Between PCOS, Oral Microbiota, and Systemic Antibody Responses [24]	Comparative observational	125 participants (PCOS with and without gingivitis, controls with and without gingivitis)	Higher pathogenic bacteria and antibody responses in PCOS gingivitis group.
Comparative Assessment of Periodontal Status in Subjects With and Without PCOS and Its Correlation With BMI [25]	Cross-sectional	30 PCOS patients, 30 controls	Higher PI, PD, and PDI in PCOS; BMI correlated with worse periodontal health.
Association Between Metabolic and Hormonal Profiles, Proinflammatory Cytokines in Saliva, and Gingival Health in Adolescent Females with PCOS [26]	Observational	31 PCOS adolescents, 28 controls	Elevated IL-6, IL-1β, TNF-α in PCOS saliva; inverse correlation of testosterone with GI.
Comparison of Prevalence of Periodontal Disease in Women with PCOS and Healthy Controls [27]	Cross-sectional	98 PCOS patients, 98 controls	Higher prevalence of periodontal disease; worse CAL and PD in PCOS (*p* < 0.05).
Evaluation of Periodontal Status in Women with PCOS Versus Healthy Women: A Cross-Sectional Study [28]	Cross-sectional	Women with PCOS vs. healthy controls	Worse GI and PD in PCOS, correlated with androgen levels.
Gingival Inflammation and Leukocyte–Endothelial Cell Interactions in Women with PCOS [29]	Observational	PCOS patients, mechanistic analysis	Increased inflammation markers, heightened neutrophil activity linked to PCOS.

**Table 2:** Summary and explanation by table of the main studies in this meta-analysis.

Title	Inclusion/Exclusion Criteria	Primary Outcome Measured
Impact of Body Mass Index and PCOS Subtypes on Periodontal Health in Chinese Women with PCOS and Periodontitis [21]	Inclusion: PCOS diagnosis based on Rotterdam criteria, periodontitis based on 2018 International Classification. Exclusion: BMI ≥ 30 kg/m^2^, systemic diseases, smoking, medications.	GBI, probing depth, hormonal levels, BMI subgroups.
Subgingival Microflora in Adolescent Females with PCOS and Its Association with Oral Hygiene, Gingivitis, and Selected Metabolic and Hormonal Parameters [22]	Inclusion: Adolescent females, PCOS based on Rotterdam criteria. Exclusion: Systemic diseases, medications, orthodontic appliances, untreated non-vital teeth.	Subgingival bacterial counts, PLI, GI, hormonal profiles.
Periodontal Disease in Polycystic Ovary Syndrome [23]	Inclusion: PCOS diagnosis per Rotterdam criteria. Exclusion: Smokers, systemic diseases, drug use, BMI ≥ 27 kg/m^2^.	PD, GI, BOP%, GCF MPO/NO.
Association Between PCOS, Oral Microbiota, and Systemic Antibody Responses [24]	Inclusion: PCOS diagnosis per Rotterdam criteria, gingivitis diagnosis based on bleeding sites. Exclusion: BMI > 30 kg/m^2^, medications affecting periodontal status, systemic diseases.	Bacterial counts, antibody responses, PD, BOP.
Comparative Assessment of Periodontal Status in Subjects With and Without PCOS and Its Correlation With BMI [25]	Inclusion: PCOS diagnosis, minimal 16 natural teeth. Exclusion: Pregnant women, smokers, periodontal treatment in last 6 months.	PI, PD, PDI, BMI.
Association Between Metabolic and Hormonal Profiles, Proinflammatory Cytokines in Saliva, and Gingival Health in Adolescent Females with PCOS [26]	Inclusion: PCOS diagnosis, newly diagnosed, untreated cases. Exclusion: Systemic diseases, antibiotics in last 6 months, orthodontic appliances.	IL-6, IL-1β, TNF-α, GI, salivary testosterone.
Comparison of Prevalence of Periodontal Disease in Women with PCOS and Healthy Controls [27]	Inclusion: PCOS diagnosis, healthy controls. Exclusion: BMI > 25, systemic diseases, periodontal treatment in last 6 months.	CAL, PD, prevalence of periodontal disease.
Evaluation of Periodontal Status in Women with PCOS Versus Healthy Women: A Cross-Sectional Study [28]	Inclusion: PCOS diagnosis, age-matched controls. Exclusion: Smokers, systemic diseases.	GI, PD, hormonal levels.
Gingival Inflammation and Leukocyte–Endothelial Cell Interactions in Women with PCOS [29]	Inclusion: PCOS diagnosis, mechanistic focus. Exclusion: Systemic diseases, medications influencing inflammation.	Neutrophil activity, GCF inflammation markers.

**Table 3:** The GRADE of evidence of this systematic review.

Certainty Assessment							№ of Patients		Effect		Certainty	Importance
№ of Studies	Study Design	Risk of Bias	Inconsistency	Indirectness	Imprecision	Other Considerations	[Intervention]	[Comparison]	Relative (95% CI)	Absolute (95% CI)		
New outcome												
9	Non-randomized studies	Not serious	Not serious	Not serious	Not serious	All plausible, residual, and confounding results would suggest a spurious effect, while no effect was observed.	331/229 (144.5%)	313/229 (136.7%)	Not estimable		⨁⨁⨁⨁ High	Important
								0.0%				

**CI**—confidence interval; ⨁: high

## Data Availability

No new data were created or analyzed in this study.

## Abbreviations

PCOS	polycystic ovary syndrome
PD	periodontal disease
